# Discovery of the Inhibitory Effect of a Phosphatidylinositol Derivative on P-Glycoprotein by Virtual Screening Followed by *In Vitro* Cellular Studies

**DOI:** 10.1371/journal.pone.0060679

**Published:** 2013-04-09

**Authors:** Xavier Lucas, Silke Simon, Rolf Schubert, Stefan Günther

**Affiliations:** 1 Pharmaceutical Bioinformatics, Institute of Pharmaceutical Sciences, Albert-Ludwigs-University, Freiburg, Germany; 2 Pharmaceutical Technology and Biopharmacy, Albert-Ludwigs-University, Freiburg, Germany; University of Edinburgh, United Kingdom

## Abstract

P-glycoprotein is capable of effluxing a broad range of cytosolic and membrane penetrating xenobiotic substrates, thus leading to multi-drug resistance and posing a threat for the therapeutic treatment of several diseases, including cancer and central nervous disorders. Herein, a virtual screening campaign followed by experimental validation in Caco-2, MDKCII, and MDKCII *mdr1* transfected cell lines has been conducted for the identification of novel phospholipids with P-gp transportation inhibitory activity. Phosphatidylinositol-(1,2-dioctanoyl)-sodium salt (8∶0 PI) was found to significantly inhibit transmembrane P-gp transportation *in vitro* in a reproducible-, cell line-, and substrate-independent manner. Further tests are needed to determine whether this and other phosphatidylinositols could be co-administered with oral drugs to successfully increase their bioavailability. Moreover, as phosphatidylinositols and phosphoinositides are present in the human diet and are known to play an important role in signal transduction and cell motility, our finding could be of substantial interest for nutrition science as well.

## Introduction

Around 5% of the human genes are involved in lipid synthesis and regulation. Not surprisingly, these biomolecules have crucial biological functions: They are the primary component of the cellular membrane, can act as first and second messengers in signal transduction and molecular recognition processes, and serve as energy storage [1]. Moreover, they modulate the activity of transporter membrane proteins, like the (ATP)-binding cassette P-glycoprotein (P-gp). Substrates transported by P-gp are highly diverse, mostly from amphipathic and neutral or weakly basic nature, and including compounds ranging in molecular weight from less than 200 Da to almost 1900 Da [2]. As a result, the transporter is capable of effluxing a broad range of cytosolic xenobiotic substrates, thus affecting the absorption of several types of drugs and eventually leading to multi-drug resistance (MDR) [3], [4]. In fact, there is strong evidence of the role of P-gp in the development of diseases and addictions [5]–[8], posing a real threat for their therapeutic treatment. During the last decades, inhibition of P-gp has been used as a mechanism to combat MDR in cancer therapy, and many small molecules modulating its activity have been described [9], [10]. Examples of such molecules include the calcium channel blocker verapamil, natural products like quinidine and the immunosuppressive agent cyclosporine A, and other surfactants and amphiphilic substances [11]–[13]. Even though the exact molecular mechanisms have yet to be completely elucidated, P-gp inhibition is mainly due to tightly binding and blocking the transmembrane substrate binding pockets or by inhibition of the ATPase activity of the cytoplasmic nucleotide-binding domain [2], [14]. Inhibition can also be induced by allosteric modulation with substances binding to non-substrate binding sites within the transmembrane regions or by interaction with the P-gp surrounding cell membrane [15]–[17]. Recently, the modulation of ATPase activity and transport inhibition of several phospholipids, including phosphatidylcholine (PC), -ethanolamine (PE), -glycerol (PG), and -serine (PS) derivatives, has been studied at the cellular level. Two phosphatidylcholine derivatives, namely 8∶0 PC and 10∶0 PC, have been found to significantly reduce ATPase activity and inhibit membrane transport of P-gp substrates [18]. Phosphatidylinositols and their phosphorylated adducts play a key role in many biological processes, e.g. signal transduction and cytokinesis [19]–[22]. However, their role in substrate transportation by P-gp has not yet been studied.

**Figure 1:**
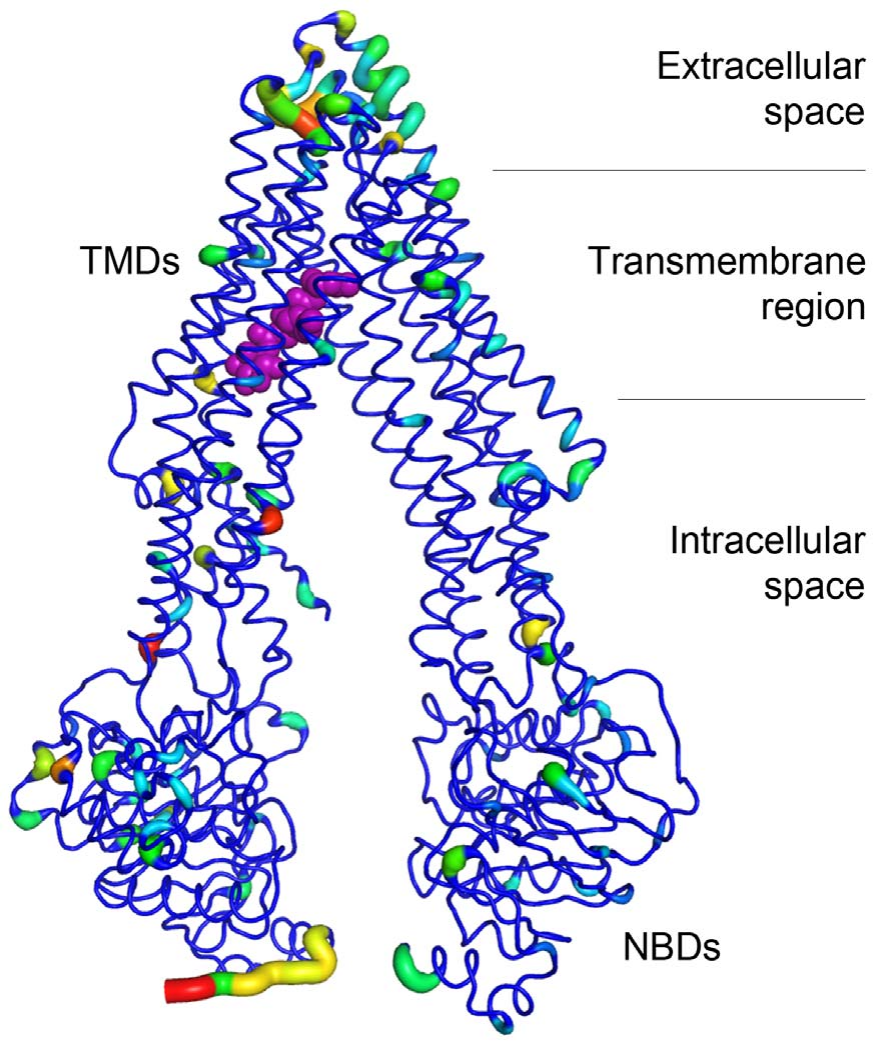
Homology model of human P-gp used in the molecular docking experiment. Tube width and residue colouring depict sequence conservation, ranging from thin and blue (identity) to wide and red (null conservation). Compound 8∶0 PI upon binding to the substrate channel is shown in purple spheres. Location of P-gp in the cellular membrane and the TMDs and NBDs are indicted.

P-glycoprotein consists of two α-helical transmembrane domains (TMDs), and two nucleotide binding domains (NBDs) (Fig. 1). The TMDs contain the substrate-binding sites and the translocation conduit [23]. Different binding sites for xenobiotics or drugs have been described, as well as the possibility of allocating two molecules simultaneously [24], [25]. Drug transport by P-gp is driven by a switch between two main conformational states of the NBD dimer: ATP binding to the drug-binding competent state induces a rotation of the NBDs and adoption of a close conformation, whereas ATP hydrolysis leads back to the open conformation of the dimer. The close conformation mediates substrate translocation in the TMDs drug-binding sites, thus triggering the release of the substrate to the extracellular face of the membrane [26]. The elucidation of the 3D structure of mouse P-gp in the drug-binding competent state allows for the virtual screening and rational design of modulators of human P-gp [27]. In the present manuscript, a virtual screening experiment followed by *in vitro* cellular assays was carried out for the identification of novel phospholipids with ability to modulate transmembrane transportation by P-gp.

## Materials and Methods

### Target Generation

Three crystal structures of P-gp in the drug-binding competent conformation from *Mus musculus* are available so far (PDB IDs: 3G5U, 3G60, and 3G61 [27]). As they show a low structural difference [28], we utilized the best resolved 3G5U *apo*-P-gp structure as a template to generate a homology model for the human transporter (Fig. 1, protein model included in PDB format as Supplementary Material), using the software Prime 2.2 (Schrödinger, LLC, New York, USA). Model quality was assessed using the Swiss-Model server [29]. The whole inner intermembrane region of the transporter was subjected to a computational molecular binding analysis. The quality of the model was assessed by performing molecular docking experiments with the known inhibitors Elacridar and Verapamil (XP scores of −10.9 and −9.9, respectively). We further used experimental Caco-2 results to study the prediction capabilities of our docking model, and observed 70% accuracy in the discrimination between active and inactive phospholipid molecules (Table S2).

### Library Preparation

We used an in-house instance of the workflow management system Galaxy [30] to generate a ligand library containing 178 distinct phospholipids with chain length ≥6 of the phosphatidylcholine (PC), -ethanolamine (PE), -glycerol (PG), -inositol (PI), -serine (PS) and phosphatidic acid (PA) compound classes by performing substructure searches (query scaffolds shown in Table S1) on the Dictionary of Natural Products [31], ChEMBL [32], and ZINC [33] libraries. Those compounds were duly processed using LigPrep 2.4 (Schrödinger, LLC, New York, USA) at pH 6.5±2.0, for the sake of consistency with the buffered experimental validation process.

### Docking Experiment

Molecular docking calculations on the human transporter model were performed with Glide 5.6 (Schrödinger, LLC, New York, USA) using the eXtra Precision (XP) docking algorithm with extended sampling, to ensure an accurate treatment of the inherent flexibility of mainly large, unsaturated lipid chains. Statistical analysis of the top 18 molecules (10% of the library, see Results and Discussion section and Fig. S1) led to the selection of 4 candidate phospholipid derivatives that were subsequently purchased for further experimental analysis.

### Materials

Phosphatidylinositol-1,2-dioctanoyl-sodium salt (PtdIns 8∶0-Na, 8∶0 PI) and phosphatidylinositol-(5)-P_1_-1,2-dihexanoyl-sodium salt (PtdIns P_1_ 6:0-Na, 6∶0 PIP_1_) were purchased from Cayman Chemical Company, Inc. (Ann Arbor, Michigan, USA). 1-Stearoyl-2-oleoyl-*sn*-glycero-3-phosphate-sodium salt (18∶0/18∶1 PA) and 1,2-dimyristoyl-*sn*-glycero-3-phosphate-sodium salt (14∶0 PA, DMPA) were purchased from Avanti Polar Lipids, Inc. (Alabaster, Alabama, USA).

### Statistical Analysis of the *in vitro* Assays

We applied One-Way ANOVA combined with Tukey and Levene’s test using the OriginPro 8.1G (Origin Lab) software to determine significant effects of lipid formulations compared to positive (verapamil) or negative control samples (buffer). Further details on the statistical methods along with the experimental setting have been already described elsewhere [18].

## Results and Discussion

**Figure 2:**
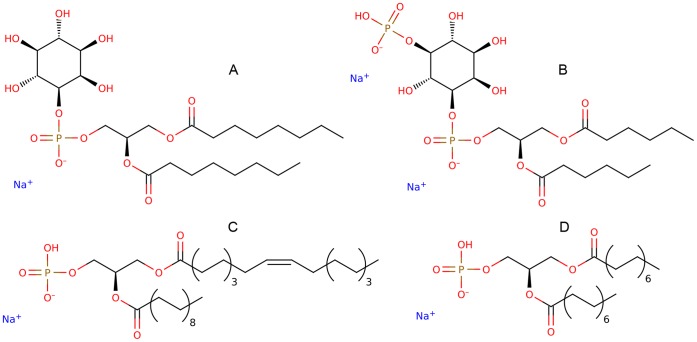
Candidate compounds selected for experimental validation. a) Phosphatidylinositol-1,2-dioctanoyl-sodium salt, 8∶0 PI or PtdIns 8∶0-Na. b) Phosphatidylinositol-(5)-P_1_-1,2-dihexanoyl-disodium salt, 6∶0 PIP_1_ or PtdIns P_1_ 6:0-Na. c) 1-Stearoyl-2-oleoyl-*sn*-glycero-3-phosphate-sodium salt, 18∶0/18∶1 PA. d) 1,2-Dimyristoyl-*sn*-glycero-3-phosphate-sodium salt, 14∶0 PA.

Herein, we performed a virtual screening campaign for the discovery of novel phospholipids with cell transport activity (see Methods). Among the possible mechanisms described for P-gp inhibition [34], we focused on molecules that would either sterically block, allosterically modulate, or compete with other substrates of interest; therefore we took into consideration the TMDs of the transporter. Figure 1 depicts the homology model built for human P-gp. The transmembrane region of the transporter, subjected to the molecular docking experiment, shows a very high sequence identity. Regions of the protein exposed to the intra- and extracellular spaces present a lower conservation. This model exhibited 70% accuracy in the discrimination between active and inactive phospholipids in docking studies (Table S2). Thus, a total of 178 distinct molecules of the PA, PC, PE, PG, PI, and PS compound classes were virtually tested on the substrate-binding domain of the transporter. Interestingly, phospholipids of the previously unreported PI class very significantly accumulated on the top 10% of the screening results (see Table 1), suggesting a preference of the transporter to interact with phosphatidylinositol derivatives. From those 18 top-ranked molecules, PA derivatives accumulated significantly but to a lesser extent. Docking poses of the other phosphatidyl classes did not accumulate significantly within the top scoring range (XP scores from −12 to −15). These findings led to the selection of two purchasable candidate compounds of each of the PI and PA classes for further experimental *in vitro* testing, namely 8∶0 PI, 6∶0 PIP_1_, 18∶0/18∶1 PA, and 14∶0 PA (Fig. 2).

**Table 1:** Phospholipid class distribution for the *in silico* results.

Phospholipid class	Representatives in top 10%	Total amount in library	*p* 1
**PA**	5	28	**0.04** *
**PC**	2	58	**0.97**
**PE**	1	29	**0.83**
**PG**	0	9	**0.63**
**PI**	9	21	**1.4·10^−6^** ***
**PS**	1	18	**0.57**
**Others** 2	0	15	**0.81**
Total	18	178	–

Classification in PA, PC, PE, PG, PI, and PS (phosphatidic acid, phosphatidylcholine, -ethanolamine, -glycerol, -inositol, and -serine, respectively). The statistical significance *p* of each observation is shown.

1 Probability *p* computed using the hypergeometric distribution.

2 Due to substructure-based searches, some of the phospholipid substitutions clearly differentiate from the main scaffolds.

* indicates a statistical significance *p*<0.05.

*** indicates a statistical significance *p*<0.001.

The binding mode obtained for compound 8∶0 PI in the substrate binding pocket of human P-gp indicates that its polar head group entangles in hydrogen-bond interactions with Asn721, Gln725, and Gln990 (Fig. S2). These polar residues define a positively charged region on the protein surface (Fig. S3, coloured in blue) that surrounds the acidic and inositol moieties of the lipid, while the aliphatic fatty acid chains remain in lipophilic cavities of the pocket (mainly coloured in white or pale). Indeed, most of the pocket is of hydrophobic nature, accounting for the promiscuous behaviour of the transporter [2]. Notably, the analysis of the location of the phosphate group in the rest of the top-ranked 18 molecules allowed for the identification of a favoured region for phosphate positioning in a cavity defined by the TM7 and TM12 α-helixes, in the asymmetric protein’s substrate pocket (Fig. S3, [27]). The phosphate group of 8∶0 PI precisely locates in the identified favoured region.

### Characterisation of the Lipid Formulations by DLS and Cryo-TEM

Depending on their structure, phospholipids can occur in a wide range of aggregation states when dispersed in an aqueous medium: dissolved as monomers, aggregated to small disordered structures, or as clearly defined vesicles, i.e. liposomes [18]. A key factor is the chain length of the fatty acid residues, since chains shorter than 6–8 carbon atoms hamper vesicle formation due to the decreased hydrophobic interactions within the lipid bilayer. Another influence is exerted by the polar head group: Charged structures induce strong repulsive forces between lipid monomers, leading to unstable aggregates, if any.

**Figure 3:**
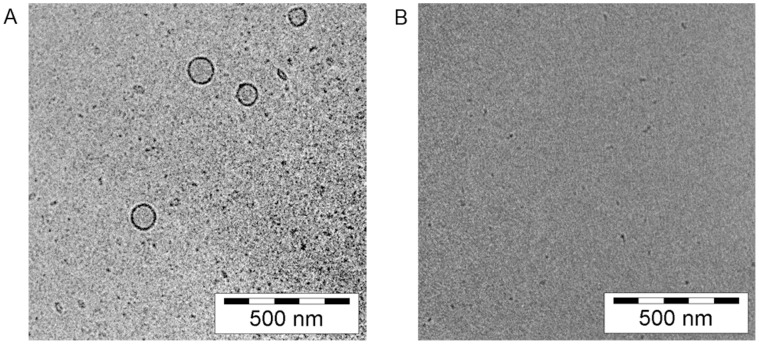
Cryo-TEM screenshots of selected phospholipid formulations. a) 18∶0/18∶1 PA vesicles in demineralised water, prepared via film method and ultra-sonication (15 min; 30 W); lipid conc. = 4.3 mM. b) 8∶0 PI ( = PtdIns 8∶0-Na) in demineralised water, prepared via film method and extrusion through polycarbonate membranes (21×80 nm pore size); lipid conc. = 1.0 mM.

This circumstance could be also confirmed with Cryo-Transmission Electron Microscopy (Cryo-TEM) for the derivatives tested in the current study (Fig. 3). The long-chained 18∶0/18∶1 PA formed vesicles in demineralised water, but of a very broad size distribution (Table S3). Given that the tested compounds were formulated as sodium-salts, the negatively charged phosphate group might have prevented intensive vesicle formation due to intermolecular electrostatic repulsive forces. In the case of PI derivatives, presenting much shorter carbon chains in addition to the negatively charged head group, the liposomal aggregation is rather unlikely. Indeed, this hypothesis is in agreement with Dynamic Light Scattering (DLS) measurements (Table S3), as the PDI values exceeded the limit of 0.8, therefore impeding meaningful size determinations. Figure 3b verifies that a 8∶0 PI ( = PtdIns 8∶0-Na) formulation of 1.0 mM in demineralised water contained neither vesicles nor aggregates. Thus, we concluded that 8∶0 PI occurred as a solubilized monomer.

### Transport Studies across Caco-2 Monolayers

**Figure 4:**
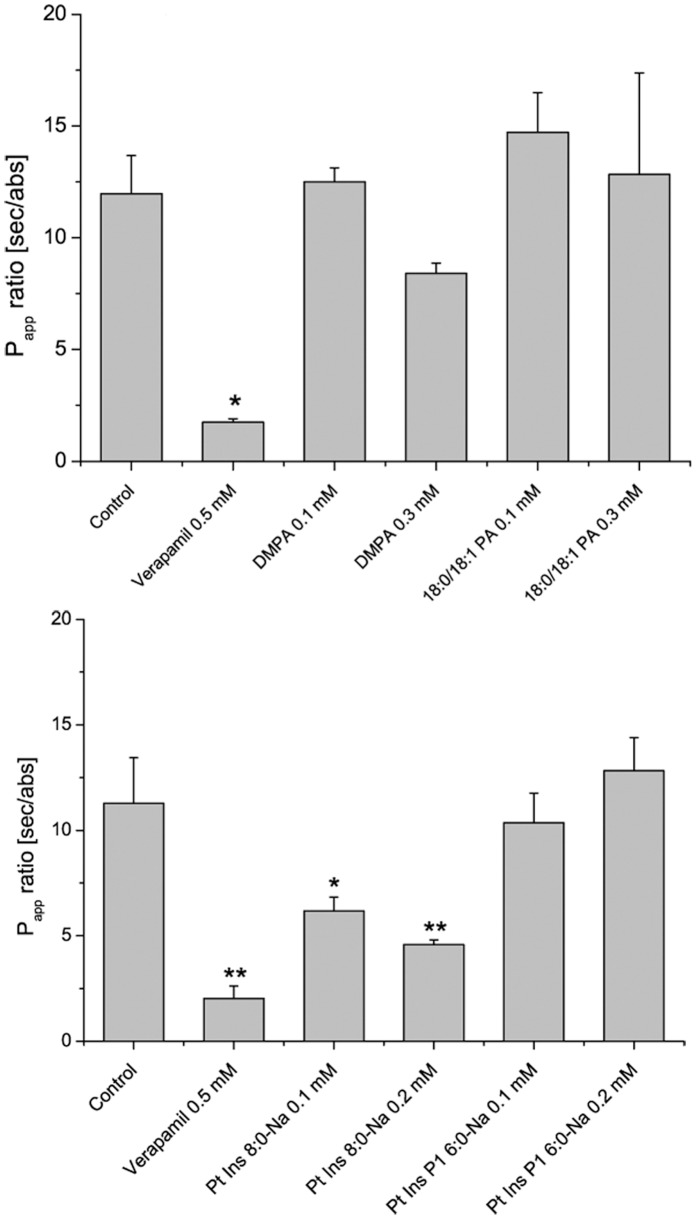
Apparent permeability (*P*_app_) ratio of digoxin transport in Caco-2 cells. Digoxin flux was measured in pure HBSS pH 6.5 (Control) and after 30 min of pre-incubation with verapamil or liposomal formulations of phosphatidic acid and phosphatidylinositol derivatives in buffer (top and bottom, respectively); n = 3; results are given as mean+standard deviation (*: significance level: 0.05 compared to control; **: significance level: 0.01 compared to control).

We studied the interference of our candidate compounds with the transport capabilities of the P-gp substrate digoxin, by measuring the differential flux of the transport marker across a cell layer, due to significant changes in secretion or absorption (Fig. 4 and Table S4). As transport medium we chose isotonic hank’s balanced salt solution (HBSS) pH 6.5. Digoxin transport (1 µM; partially ^3^H-labelled) in untreated human colon carcinoma (Caco-2) cell layers was distinctly asymmetric, the secretory flux exceeding roughly by 12-fold the absorptive one (control). This accounted for the distinct expression of the efflux transporter, especially as the substrate flux was nearly equalized after pre-incubation with the established P-gp inhibitor verapamil. Caco-2 cells are a well-established biopharmaceutical tool to investigate absorption processes across the human intestinal mucosa; therefore, these transport studies are of high importance to predict oral bioavailability enhancement due to P-gp inhibition [35], [36].

In the case of phosphatidic acid derivatives (Fig. 4a), no alteration of digoxin transport was observed after pre-incubation with 14∶0 PA at 0.1 mM. Increase of the lipid amount led to a reduction of the apparent permeability (*P*_app_) ratio value by 30%. However, this increase in concentration provoked a drop to 63% in transepithelial electrical resistance (TEER), indicating a disruption of the cell monolayer. The unsaturated PA derivative, 18∶0/18∶1 PA, did not show any potency in inhibition of digoxin transport, even at a high concentration of 0.3 mM. These observations do not agree with the *in silico* predictions. However, docking studies were conducted with the respective lipid monomer and any aggregation, such as vesicle formation or other possible polymeric interferences, was not taken into account. Since both PA derivatives present long, mainly saturated fatty acid chains, they tend to form lipid aggregates, as depicted in Fig. 3a [37]. Previous studies have shown that phospholipids might interact with P-gp preferentially in their monomeric state [18], thus compounds with a low monomeric concentration might not be able to cause observable transporter inhibition.

Figure 4b features the modulation produced in the digoxin transportation by human P-gp in Caco-2 studies after pre-incubation with our candidate phosphatidylinositol derivatives. Interestingly, 8∶0 PI ( = PtdIns 8∶0-Na) induced a significant (45%) reduction of *P*_app_ ratio at 0.1 mM combined with a TEER value within the acceptable limit. A 2-fold increase in lipid concentration promoted a stronger inhibition of transportation, but at the expense of destroying the cell integrity nearly completely. No effect on transportation was observed after pre-incubation with 6∶0 PIP_1_.

The striking and singular efficacy of 8∶0 PI might be related to its caprylic moiety, also found in 8∶0 PC, which was recently identified as well as active in inhibiting P-gp transportation [18]. Moreover, this short fatty acid chain increases the critical micelle concentration of the lipid, leading to a comparably high monomer amount in the formulation (Fig. 3b). Another key aspect of the compound’s activity could be explained by analysing its binding pose (Fig. S2). Axial hydroxyl groups at positions *C*_2_ and *C*_3_ of the *myo*-inositol moiety of 8∶0 PI are involved in hydrogen-bond interactions with Gln725, accounting for specific and unique interactions that would increase ligand affinity. The location of the compound upon binding, as shown in Figure 1, indicates that 8∶0 PI inhibits P-gp apical transportation by tightly binding and blocking its TMDs.

### Calcein Accumulation Assay (CAA)

After obtaining indications for P-gp modulation activity for 8∶0 PI in Caco-2 studies, it was further subjected to CAA, which detects the P-gp-dependent intracellular accumulation of the fluorescent dye calcein. This assay was performed in parallel in a parental wild type (wt) Madin Darby canine kidney (MDCK) II cell line with basal P-gp expression and its corresponding P-gp-overexpressing strain, which is stably transfected with human *mdr1*. In this test, the degree of P-gp-inhibition directly correlates with the entrapped amount of calcein and, thus, the intensity of intracellular fluorescence. Given that P-gp expression level is the unique difference between both MDCKII strains, different increases in fluorescence intensity in the *mdr1* transfected and wt clearly indicate true P-gp modulation [38].

**Figure 5:**
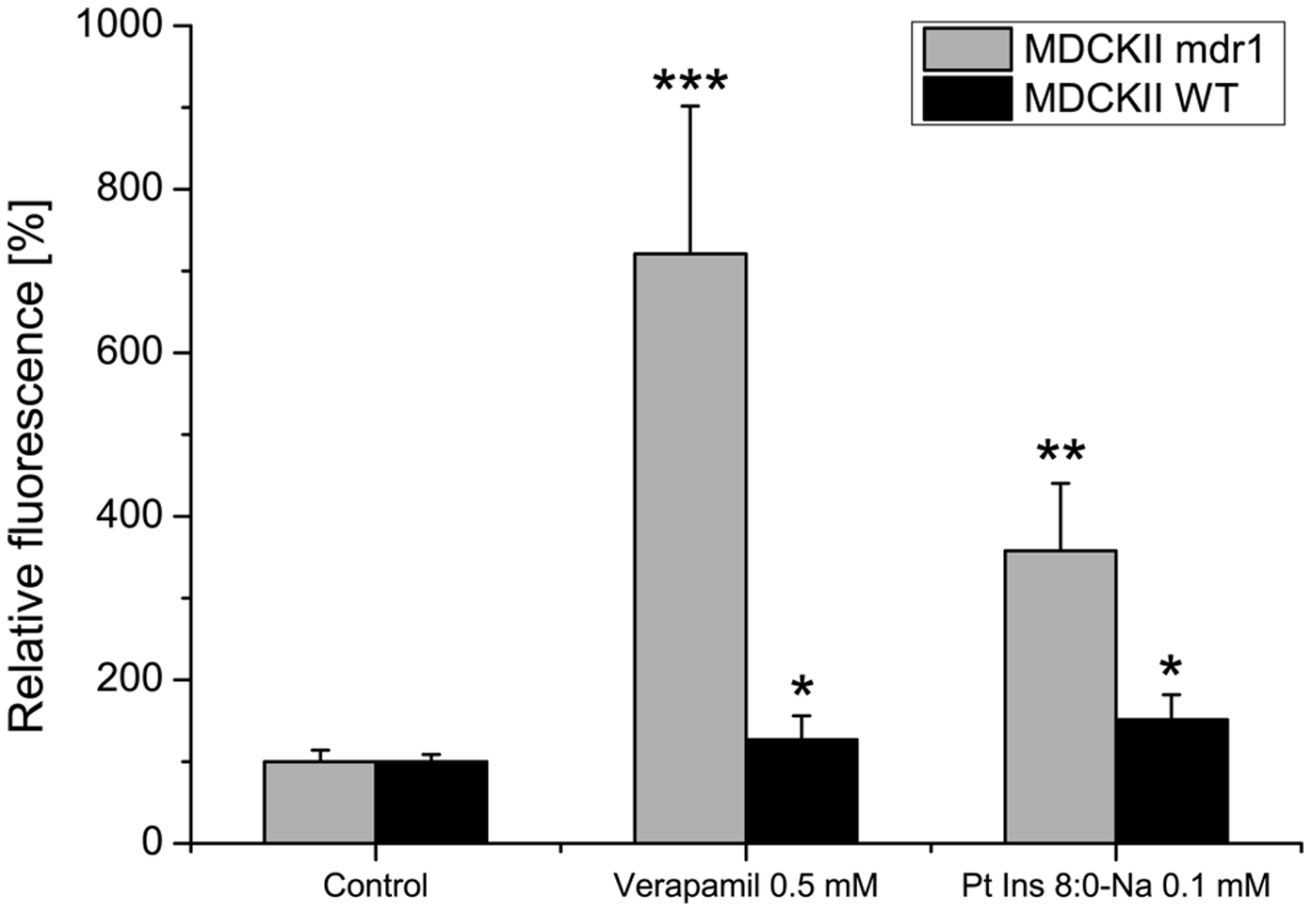
Calcein Accumulation Assay results. Relative intracellular calcein fluorescence signal in MDCKII mdr1 and wild type (wt) cells (in grey and black, respectively) after pre-incubation with verapamil or 8∶0 PI ( = PtdIns 8∶0-Na) in HBSS with respect to the control (100% RF; calcein-acetoxymethylester in HBSS); n = 6; results are given as mean+standard deviation (*: significance level: 0.05 compared to control; **: significance level: 0.01 compared to control; ***: significance level: 10^−3^ compared to control).

Figure 5 summarizes the results of CAA using HBSS pH 6.5 as assay buffer. The fluorescence intensities of each cell line treated solely with the calcein-derivative were set to 100% ( = control). With the other intensity values referring to this control, an enhancement due to P-gp inhibition became visible. Incubation with the positive control verapamil (0.5 mM) led to an increase in intracellular fluorescence by 1.5-fold in wt cells and 7-fold in *mdr1*. This difference in effect verified the true impact of Verapamil on the P-gp transporter. The same effect can be observed for 8∶0 PI ( = PtdIns 8∶0-Na), inducing a 3.8-fold increase in calcein accumulation in the overexpressing strain, but only an 1.8-fold enhancement in wt.

These results indicate that 8∶0 PI exerts a reproducible-, cell line-, and substrate-independent P-gp inhibition.

### Conclusions

In the present manuscript we have conducted a virtual screening campaign for the identification of novel phospholipids capable of inhibiting cellular membrane transportation by P-gp. Compounds of the previously unstudied phosphatidylinositol class were identified as potential binders by molecular docking analysis (Table 1). The experimental validation of 4 candidate compounds, namely 18∶0/18∶1 PA, 14∶0 PA, 6∶0 PIP_1_, and 8∶0 PI, led to the discovery of the latter as an active modulator of P-gp transportation. 8∶0 PI exerted a significant transportation inhibition of the substrate digoxin in Caco-2 cells, which was subsequently unequivocally correlated to P-gp modulation in comparative MDCKII CAA studies (Fig. 4 and 5). Moreover, this compound showed a high tendency to exist as a monomer in solution (Fig. 3b), which has been described as a favoured state for P-gp inhibition [18]. Even though several possible mechanisms have been formulated for the inhibition of this transporter, our hypothesis –based on the assumptions of our theoretical model– is that 8∶0 PI modulates transportation either by steric blockade of the substrate-binding domain, by allosteric modulation or by competition with other transported substrates (Fig. 1, [34]).

All in all, the identification of compounds that inhibit P-gp transportation is particularly interesting for their implications in combating multi-drug resistance. In fact, the co-administration of P-gp inhibitors along with P-gp substrate drugs might be a promising option in pharmacological formulation development in order to overcome drug resistance. Moreover, and due to the important role of phosphatidylinositols and phosphoinositides in many biological processes, such as cell motility and signalling [19]–[22], the unexpected discovery of the P-gp inhibitory properties of 8∶0 PI may account for a natural mechanism of regulation of cellular membrane function and xenobiotic efflux. Currently, several biological functions of exogenous PI are known, including inhibition of vascular endothelial growth factor (VEGF)-induced angiogenesis, inhibition of low concentrations of amyloid *β* protein induced degeneration, and promotion of cholesterol transport and excretion eventually inducing anti-obesity response in mice [39]–[43]. As phospholipids are a common constituent of human diet –for example, phosphoinositols are found in significant amounts in soybean–, it becomes important to further characterise and analyse their role in transmembrane transportation, and study their possible effects in *in vivo* models for combating MDR.

## Supporting Information

Figure S1 **Distribution of XP scores in the molecular docking experiment.** The vertical line at x = 18 indicates the top 10% of the results.
(TIF)

Figure S2 **Inter-molecular hydrogen-bond interactions (as yellow dashed lines) present in the predicted pose of 8∶0 PI (in purple sticks) in the substrate-binding pocket of human P-gp (in grey cartoon).** Interacting residues (Asn721 and Gln725 from TM7 α-helix, and Gln990 from TM12) are highlighted.
(TIF)

Figure S3 **Location of the phosphorus atom of the phosphatidyl group in the top-ranked 18 compounds upon docking in the substrate binding pocket of human P-gp, depicted as coloured spheres (the colouring scheme identifies the compound class, see legend and Table S1 for details).** For reference purposes, the binding pose of 8∶0 PI is shown in thin purple sticks. Protein surface coloured by electrostatic potential: positive in blue, neutral in white, and negative in red.
(TIF)

Table S1 **Representation of the chemical scaffolds used as query for the substructure searches (PA, PC, PE, PG, PI, and PS stand for phosphatidic acid, phosphatidylcholine, -ethanolamine, -glycerol, -inositol, and -serine, respectively).**
(DOCX)

Table S2 **Comparison of experimental and predicted values used for validation of the theoretical model.** Their relative *P*_app_ ratio, XP score, and the consistency of predicted and experimental results are shown.
(DOCX)

Table S3 **Hydrodynamic diameter and polydispersity index (PDI) of the applied phospholipid formulations measured via Dynamic Light Scattering.** Values given as mean ± standard deviation from three measurements (21 single runs).
(DOCX)

Table S4 ***P*** **_app_ ratio of tested compounds (referred to the control value) and impact on TEER in Caco-2 transport studies.**
(DOCX)

File S1 **Homology model of the human P-gp transporter.**
(PDB)
