# Supplementary figures and images for: Discovery of the Inhibitory Effect of a Phosphatidylinositol Derivative on P-Glycoprotein by Virtual Screening Followed by In Vitro Cellular Studies
Source: PLoS One. 2013 Apr 9;8(4):e60679. doi: 10.1371/journal.pone.0060679 (PMC3621910; doi:10.1371/journal.pone.0060679)

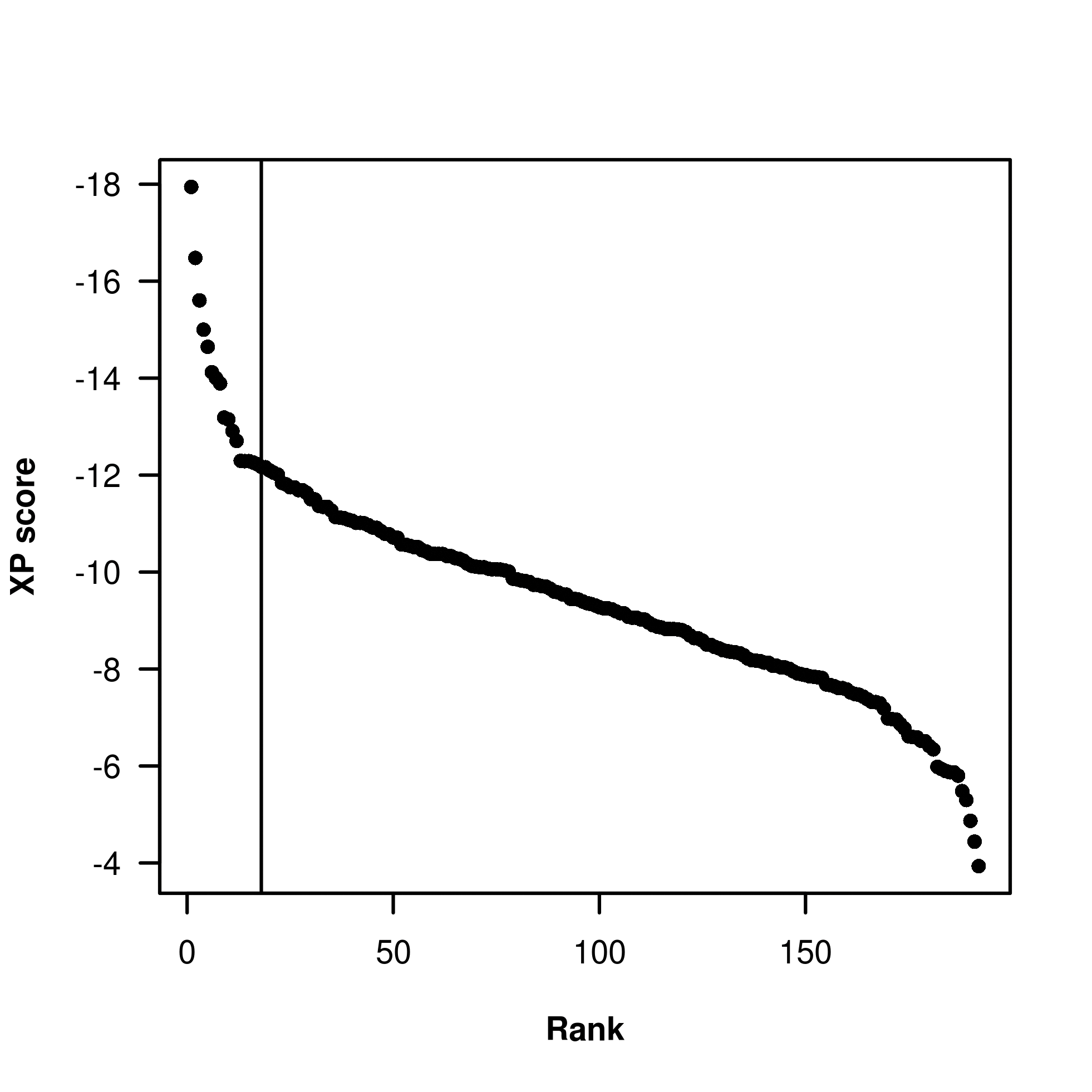

Supplement: Figure S1 — Distribution of XP scores in the molecular docking experiment. The vertical line at x = 18 indicates the top 10% of the results. (TIF) [file pone.0060679.s001.tif]

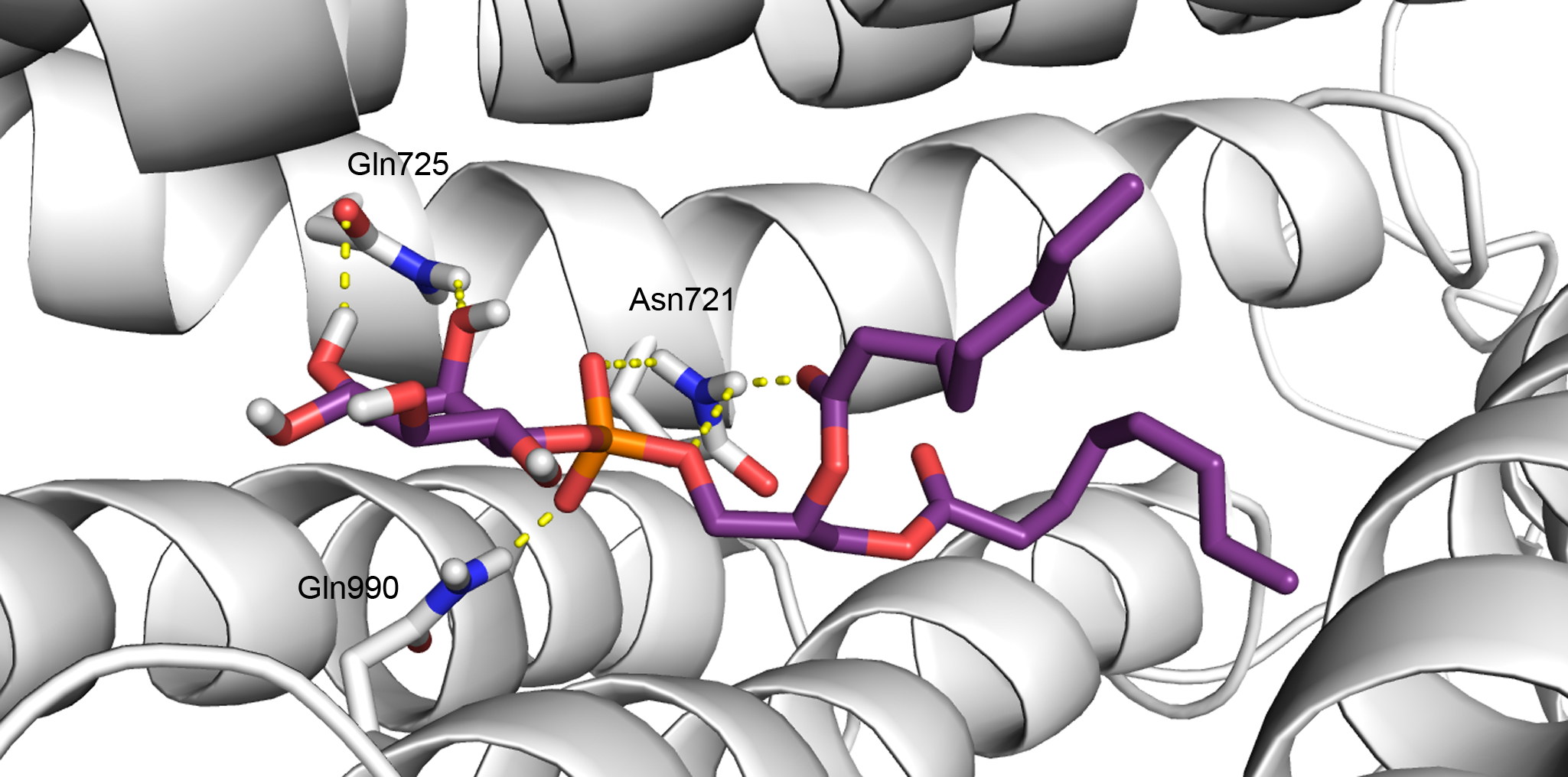

Supplement: Figure S2 — Inter-molecular hydrogen-bond interactions (as yellow dashed lines) present in the predicted pose of 8∶0 PI (in purple sticks) in the substrate-binding pocket of human P-gp (in grey cartoon). Interacting residues (Asn721 and Gln725 from TM7 α-helix, and Gln990 from TM12) are highlighted. (TIF) [file pone.0060679.s002.tif]

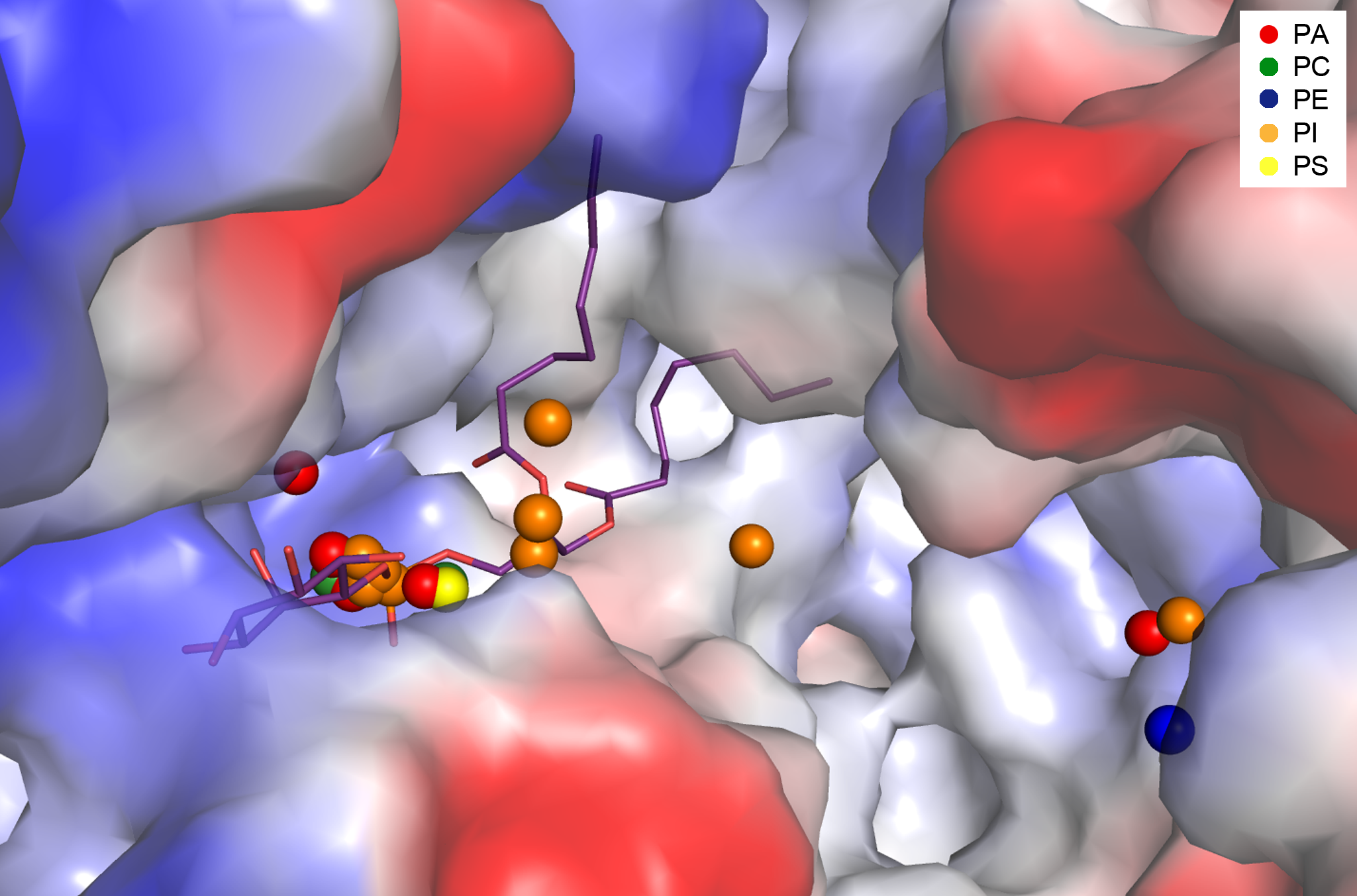

Supplement: Figure S3 — Location of the phosphorus atom of the phosphatidyl group in the top-ranked 18 compounds upon docking in the substrate binding pocket of human P-gp, depicted as coloured spheres (the colouring scheme identifies the compound class, see legend and Table S1 for details). For reference purposes, the binding pose of 8∶0 PI is shown in thin purple sticks. Protein surface coloured by electrostatic potential: positive in blue, neutral in white, and negative in red. (TIF) [file pone.0060679.s003.tif]
