# Supplementary material for: Discovery of the Inhibitory Effect of a Phosphatidylinositol Derivative on P-Glycoprotein by Virtual Screening Followed by In Vitro Cellular Studies
Source: PLoS One. 2013 Apr 9;8(4):e60679. doi: 10.1371/journal.pone.0060679 (PMC3621910; doi:10.1371/journal.pone.0060679)
Supplement: Table S1 — Representation of the chemical scaffolds used as query for the substructure searches (PA, PC, PE, PG, PI, and PS stand for phosphatidic acid, phosphatidylcholine, -ethanolamine, -glycerol, -inositol, and -serine, respectively). (DOCX) [file pone.0060679.s004.docx]

**Table S1.** Representation of the chemical scaffolds used as query for the substructure searches (PA, PC, PE, PG, PI, and PS stand for phosphatidic acid, phosphatidylcholine, -ethanolamine, -glycerol, -inositol, and -serine, respectively).

| **PA**; R = H | 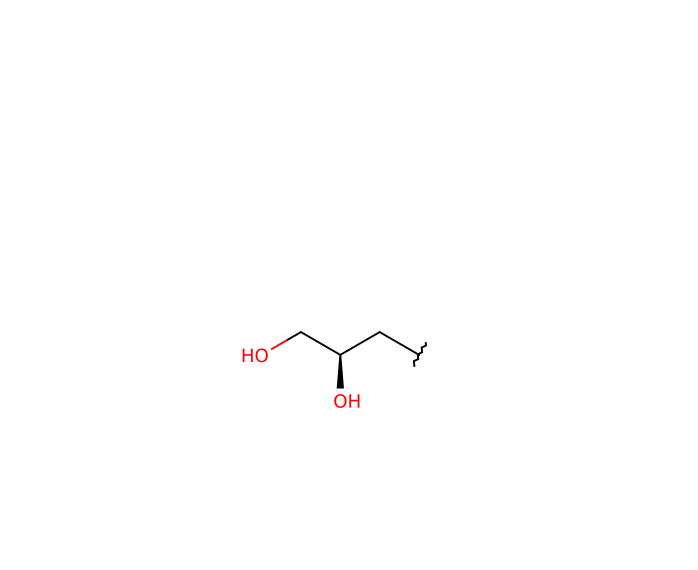  **PG**; R = | 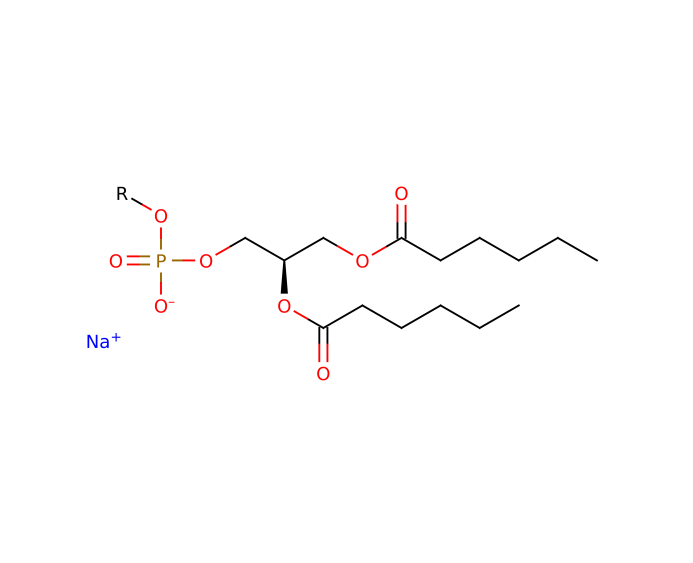 |
| --- | --- | --- |
| **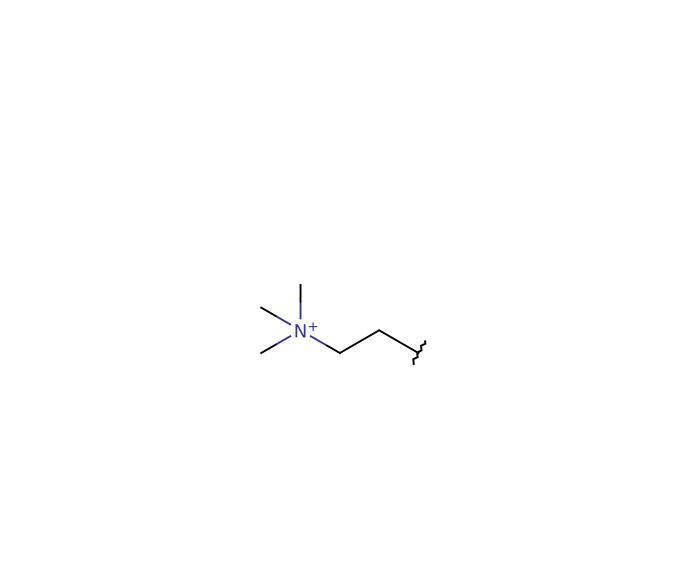PC**; R = | 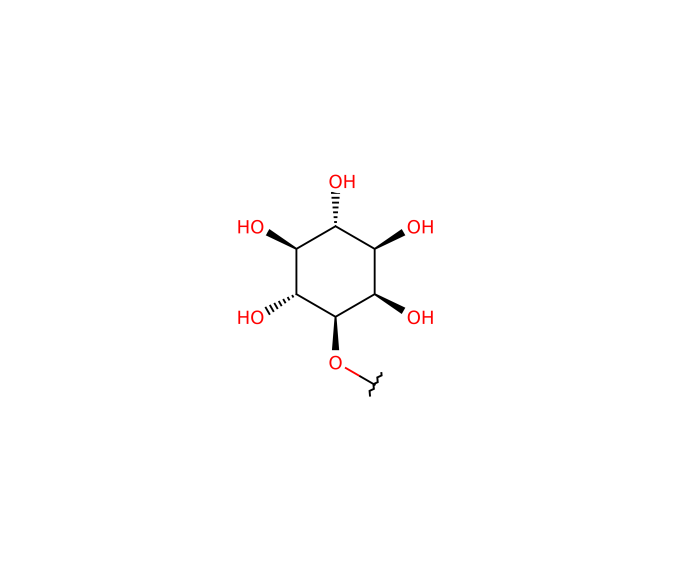**PI**; R = |  |
| 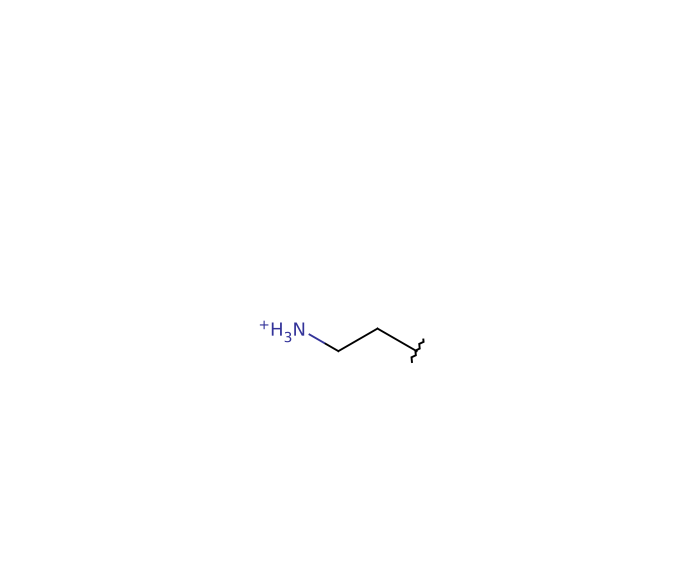**PE**; R = | 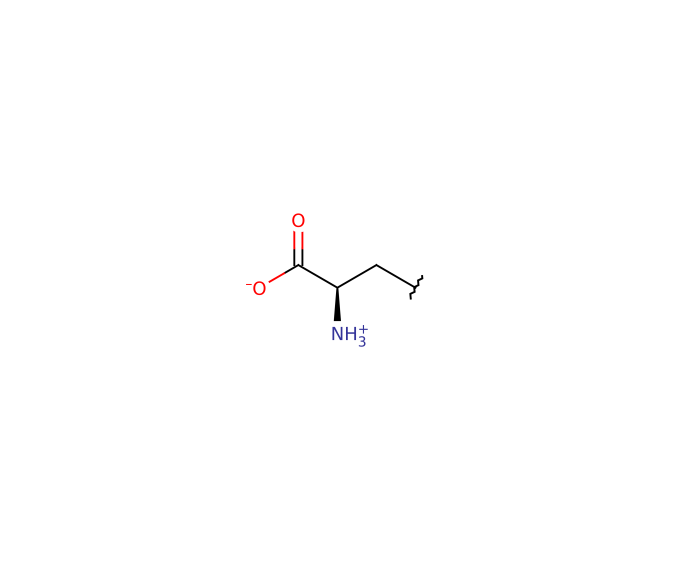  **PS**; R = |  |
