# Supplementary material for: Discovery of the Inhibitory Effect of a Phosphatidylinositol Derivative on P-Glycoprotein by Virtual Screening Followed by In Vitro Cellular Studies
Source: PLoS One. 2013 Apr 9;8(4):e60679. doi: 10.1371/journal.pone.0060679 (PMC3621910; doi:10.1371/journal.pone.0060679)
Supplement: Table S2 — Comparison of experimental and predicted values used for validation of the theoretical model. Their relative P app ratio, XP score, and the consistency of predicted and experimental results are shown. (DOCX) [file pone.0060679.s005.docx]

**Table S2.** Comparison of experimental and predicted values used for validation of the theoretical model. Their relative *P*_app_ ratio, XP score, and the consistency of predicted and experimental results are shown.

| ***Lipid*** | ***Relative P_app_ ratio [%]*** | ***XP score*** | ***Prediction-experimental consistency^2^*** |
| --- | --- | --- | --- |
| **8:0 PC** | 24.1*^,1^ | -9.1 | No |
| **10:0 PC** | 39.8*^,1^ | -10.8 | Yes |
| **12:0 PC** | 86.6^1^ | -6.8 | Yes |
| **14:0 PC** | 115.5^1^ | -4.5 | Yes |
| **16:0 PC** | 145.5 | -9.8 | Yes |
| **18:0 PC** | 113.9 | --- (no suitable binding pose identified) | Yes |
| ***trans*-18:1 PC** | 103.9^1^ | -8.9 | Yes |
| ***cis*-22:6 PC** | 39.3*^,1^ | -11.5 | Yes |
| **8:0 PG** | 99.1^1^ | -11.1 | No |
| **16:0 PG** | 69.6 | -11.4 | No |
| **18:0 PG** | 128.7 | -7.6 | Yes |
| **16:0 PE** | 116.7 | -10.9 | No |
| **10:0 PS** | 78.8^1^ | -9.4 | Yes |

*: Significant inhibition of transportation in Caco-2 and MDCKII studies.

^1^: Results previously reported (ref. 18).

^2^: Prediction was defined as “Active” if the XP score was equal or lower than that of verapamil (XP score = -9.9). Experimental validation was defined as “Active” if there was a significant transportation inhibition in Caco-2 and MDCKII studies.
