# Supplementary material for: Discovery of the Inhibitory Effect of a Phosphatidylinositol Derivative on P-Glycoprotein by Virtual Screening Followed by In Vitro Cellular Studies
Source: PLoS One. 2013 Apr 9;8(4):e60679. doi: 10.1371/journal.pone.0060679 (PMC3621910; doi:10.1371/journal.pone.0060679)
Supplement: Table S3 — Hydrodynamic diameter and polydispersity index (PDI) of the applied phospholipid formulations measured via Dynamic Light Scattering. Values given as mean ± standard deviation from three measurements (21 single runs). (DOCX) [file pone.0060679.s006.docx]

**Table S3.** Hydrodynamic diameter and polydispersity index (PDI) of the applied phospholipid formulations measured via Dynamic Light Scattering. Values given as mean ± standard deviation from three measurements (21 single runs).

| ***Compound*** | | ***Hydrodynamic diameter [nm]*** | ***PDI*** |
| --- | --- | --- | --- |
| **Phosphatidic acid derivatives** | 14:0 PA | 1761 ± 1151 | 0.745 ± 0.442 |
|  | 18:0/18:1 PA | 1006 ± 51.6 | 0.476 ± 0.014 |
| **Phosphatidylinositol derivatives** | 8:0 PI | n.a. | n.a. |
|  | 6:0 PIP_1_ | n.a. | n.a. |
