# Supplementary material for: Discovery of the Inhibitory Effect of a Phosphatidylinositol Derivative on P-Glycoprotein by Virtual Screening Followed by In Vitro Cellular Studies
Source: PLoS One. 2013 Apr 9;8(4):e60679. doi: 10.1371/journal.pone.0060679 (PMC3621910; doi:10.1371/journal.pone.0060679)
Supplement: Table S4 — P app ratio of tested compounds (referred to the control value) and impact on TEER in Caco-2 transport studies. (DOCX) [file pone.0060679.s007.docx]

**Table S4.** *P*_app_ ratio of tested compounds (referred to the control value) and impact on TEER in Caco-2 transport studies.

| ***Compound*** | ***Concentration [mM]*** | ***P*_app_ *ratio*^1^ *[%]*** | ***TEER*^2^** |
| --- | --- | --- | --- |
| **Control** | - | 100 | = |
| **Verapamil** | 0.5 | 16.25 (**) | = |
| **14:0 PA** | 0.1 | 104.50 | = |
|  | 0.3 | 70.31 | < |
| **18:0/18:1 PA** | 0.1 | 122.95 | = |
|  | 0.3 | 107.31 | = |
| **8:0 PI** | 0.1 | 54.84 (*) | =/< |
|  | 0.2 | 40.63 (**) | < |
| **6:0 PIP_1_** | 0.1 | 91.82 | = |
|  | 0.2 | 113.69 | = |

^1^ *: significance level: 0.05 compared to control. **: significance level: 0.01 compared to control.

^2^ =: constant TEER values (within the range of the control values). <: decreased TEER values (<80% of the control TEER values).
